# Validation and Clinical Adaptation of a Swedish Version of the Tonsil Outcome Inventory‐14 Questionnaire

**DOI:** 10.1002/lio2.70359

**Published:** 2026-02-18

**Authors:** Andrea Cvjetkovic, Hanna Gerhardsson, Anders Broström, Fredrik Alm, Erik Odhagen, Pia Nerfeldt, Sara Axelsson, Ola Sunnergren

**Affiliations:** ^1^ Department of Otorhinolaryngology—Head and Neck Surgery Institute of Clinical Sciences, Sahlgrenska Academy, University of Gothenburg Gothenburg Sweden; ^2^ Department of Otorhinolaryngology—Head and Neck Surgery Sahlgrenska University Hospital Gothenburg Sweden; ^3^ Department of Otorhinolaryngology Jönköping County Hospital Jönköping Sweden; ^4^ Department of Biomedical and Clinical Sciences Linköping University Linköping Sweden; ^5^ Department of Nursing, School of Health and Welfare Jönköping University Jönköping Sweden; ^6^ Department of Clinical Neurophysiology University Hospital Linköping Linköping Sweden; ^7^ Department of Health and Caring Sciences Western Norway University of Applied Sciences Bergen Norway; ^8^ School of Health Sciences Faculty of Medicine and Health, Örebro University Örebro Sweden; ^9^ Department of Otorhinolaryngology Södra Älvsborgs Hospital Borås Sweden; ^10^ Department of Otorhinolaryngology Karolinska University Hospital Stockholm Sweden; ^11^ Department of Clinical Science, Intervention and Technology (CLINTEC) Karolinska Institutet Stockholm Sweden; ^12^ Department of Otorhinolaryngology Helsingborg Hospital Helsingborg Sweden

**Keywords:** patient‐reported outcomes, quality of life, questionnaire validation, sleep‐disordered breathing, tonsillectomy

## Abstract

**Purpose:**

To translate, culturally adapt, and validate a Swedish version of the Tonsillectomy Outcome Inventory‐14 (TOI‐14) and to evaluate an extended version including three additional sleep‐disordered breathing (SDB) items (TOI‐17) among adult tonsil surgery patients.

**Methods:**

This prospective multicenter study was conducted across five Swedish ENT departments between 2022 and 2023. One‐hundred‐and‐eight adults scheduled for tonsil surgery and 84 controls without tonsil disease completed the TOI‐14, TOI‐17, and EQ‐5D VAS at baseline and 2 weeks later, and patients repeated the questionnaires 6 months after surgery. Psychometric evaluation included internal consistency, test–retest reliability, convergent and known‐groups validity, responsiveness, confirmatory factor analysis (CFA) and exploratory factor analysis (EFA).

**Results:**

At baseline, patients reported markedly higher TOI‐14 (39.5 ± 17.8) and TOI‐17 (50.7 ± 19.8) scores than controls (3.9 ± 4.5 and 4.9 ± 5.0; *p* < 0.001). Both instruments showed excellent internal consistency (*α* = 0.88 and 0.82) and high test–retest reliability (ICC = 0.89 and 0.83). Scores correlated with EQ‐5D VAS (*r* = −0.54 and −0.55; *p* < 0.001) and improved significantly 6 months postoperatively (*p* < 0.001). The CFA of the Swedish TOI‐14 showed suboptimal fit of the original four‐factor model, while EFA of the TOI‐17 supported a five‐factor solution, with the three SDB items forming a distinct, internally coherent domain (loadings 0.6–0.8).

**Conclusion:**

The Swedish TOI‐14 and TOI‐17 are valid, reliable, and responsive patient‐reported outcome measures for adults with tonsil disease. The extended TOI‐17 version adds clinically relevant coverage of sleep‐related symptoms, enhancing applicability for both clinical use and registry‐based quality assessment.

**Level of Evidence:**

III—non‐randomized controlled cohort.

## 1 Introduction

Surgical removal of the tonsils is among the most frequently performed surgical procedures under general anesthesia, for both children and adults [1]. However, uncertainties remain regarding the procedure's effectiveness for certain indications, particularly in relation to long‐term symptom relief and quality of life [2]. For example, the supporting scientific evidence is limited for some commonly cited indications such as chronic or recurrent tonsillitis [1, 3, 4], where randomized controlled trials and long‐term outcome data are sparse. Moreover, the absence of robust long‐term evidence indicates that patient selection criteria have been inconsistently applied both across nations and over time [3, 5].

To improve clinical decision‐making and resource allocation, there is a growing emphasis on incorporating preoperative patient‐reported experience measures (PREMs) and patient‐reported outcome measures (PROMs) into routine care [6]. PREMs and PROMs provide standardized, validated assessments of patients' symptoms, functional status, and quality of life, offering insights beyond traditional clinical endpoints. Their reliability and validity are critical to ensuring that they reflect meaningful and interpretable changes following interventions such as surgery.

Established in 1997 by the Swedish Association for Otorhinolaryngology—Head and Neck Surgery, the Swedish Quality Register for Tonsil Surgery (SQTS) is a national registry aimed at improving ENT care [4]. It systematically monitors surgical indications, complications, and outcomes following benign tonsil surgery. Between 2009 and 2018, longitudinal data from the SQTS showed a 15%–20% decline in the proportion of patients reporting complete symptom resolution 6 months after all types of tonsil surgery, and across most indications including recurrent tonsillitis, chronic tonsillitis, and tonsil‐related sleep‐disordered breathing (SDB) [7]. This trend could not be explained by changes in patient demographics, surgical methods, or indications. However, the absence of PREM data in the SQTS registry limits the ability to systematically evaluate both potential longitudinal changes in surgical thresholds and the impact of surgery on patient‐reported outcomes in a systematic way.

The Tonsillectomy Outcome Inventory‐14 (TOI‐14) is an instrument used to detect changes in tonsil disease related QoL preoperatively (PREM) and postoperatively (PROM). Originally developed and validated in German for patients aged ≥ 16 years with chronic tonsillitis, the instrument comprises 14 items with four domains: throat discomfort, general health, resources, and social‐psychological restrictions [8]. Original validation of the TOI‐14 demonstrated good internal consistency, test–retest reliability, and construct validity, and further identifying a clear four‐factor structure corresponding to the theoretically defined domains of throat problems, general health, resources, and social‐psychological restrictions. Additional validation in a normative German population has, however, revealed a factor structure of three components—described as physiological, psychological, and socioeconomic—explaining 73% of the total variance [9]. Despite its international relevance, TOI‐14 has not yet been translated or validated in Swedish, and no Swedish PREMs/PROMs specifically target patients with tonsillar disease.

A limitation in the current PREM/PROM landscape for tonsillar disease is the lack of instruments tailored to reflect the full spectrum of symptoms associated with tonsillar disease. For instance, tonsil‐related SDB—which includes symptoms such as snoring, fragmented sleep, and daytime fatigue—is a common indication for surgery in both adults and adolescents [2]. Yet, these symptoms are not captured in the original version of the TOI‐14. To address this gap, three additional SDB‐specific items could be easily incorporated alongside the existing 14 items to broaden the instrument's clinical relevance.

The aim of this study was to translate, validate, and clinically adapt both a Swedish version of the TOI‐14 and an extended version with three added SDB items (TOI‐17) in a Swedish cohort of tonsil surgery patients.

## 2 Materials and Methods

### 2.1 Study Design

This was a prospective observational multicenter study conducted across five ENT departments in Sweden between 2022 and 2023. The study was designed to translate and validate both a Swedish version of the TOI‐14 [8], and an extended version incorporating three SDB items (TOI‐17).

### 2.2 Study Population

One‐hundred and eight adults (≥ 16 years) eligible for tonsil surgery were consecutively recruited to a surgical cohort, also forming the case group. Patients were eligible for inclusion if they were able to read and understand Swedish and provide written consent. Exclusion criteria were malignant tonsil disease. Recruitment took place at five otorhinolaryngology departments in Sweden. Patients were recruited from routine clinical practice and all doctors at the participating departments were invited (but not obliged) to recruit patients. The initial data collection took place by the doctors during the recruitment of cases, either during the visit when the decision for surgery was made, from a surgical waiting list or at the day of surgery. This is referred to as the baseline, from which data on age, sex, and indication for surgery were collected.

Indications for surgery were defined according to the prevailing clinical practice, as no Swedish national guidelines for tonsil surgery indications exist. Consequently, the indications are not fully standardized. According to the SQTS inclusion criteria, recurrent tonsillitis was defined as three separate episodes of tonsillitis during the preceding 12 months [10]. Chronic tonsillitis was defined as chronic inflammation of the tonsils lasting at least 3 months and associated with a substantial impact on daily activities. For peritonsillitis and SDB, however, the SQTS inclusion criteria are limited in scope. Peritonsillitis is defined solely as acute or previous peritonsillitis, and tonsillectomy for SDB is defined only as obstructive breathing during sleep due to tonsillar hypertrophy. In Swedish clinical practice, symptoms such as halitosis and tonsillolithiasis are not regarded as independent indications for tonsillectomy, but rather as manifestations of chronic tonsillar disease, and these symptoms are therefore indirectly captured within existing TOI‐14 items. SDB was defined as tonsil‐related upper airway obstruction with symptoms such as snoring, disturbed sleep, or daytime fatigue.

**FIGURE 1:**
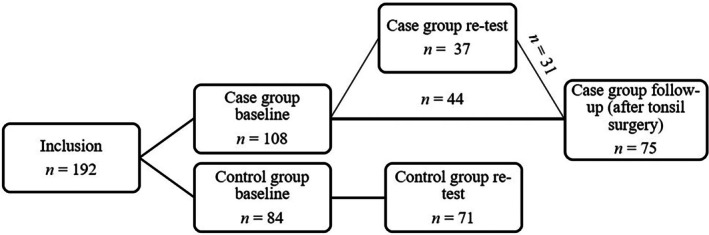
Flow chart of the study population's testing.

Eighty‐four voluntary adults (≥ 16 years) without tonsil disease were invited and recruited by the authors to inclusion in the control group. These individuals were selected through a convenience sampling method at all participating clinics, including volunteers such as department staff and medical students. Flow chart of the study population's testing is shown in Figure 1.

### 2.3 The TOI‐14 and the TOI‐17 Instruments

The TOI‐14 instrument comprises 14 items with four domains: throat discomfort (items 1–4), general health (items 5–6), resources (items 7–10), and social‐psychological restrictions (items 11–14). The items are answered by a six‐point Likert scale (0 = “no problem” to 5 = “couldn't be worse”). The score is formed by adding up the points, dividing the sum by 70 and multiplying this by 100 to give an adjusted score out of 100 (maximum). The higher the score, the poorer the tonsil disease related QoL [8, 9]. Participants were asked to answer the questions based on how things were during the past 6 months.

Based on our clinical experience and knowledge of the field, we hypothesize that the TOI‐14 is appropriate for patients with infectious and inflammatory tonsil problems such as recurrent tonsillitis, chronic tonsillitis, and peritonsillitis, but not for those with SDB. To address this limitation, three additional SDB‐related items were added to an extended version (TOI‐17), with the same six‐point Likert scale as the original instrument. These items were designed to capture key aspects of sleep‐related symptoms that are often associated with tonsillar hypertrophy or upper airway obstruction. Item 15—loud snoring during sleep—reflects the social and physical impact of loud snoring, often reported by both patients and their partners. Item 16—irregular breathing or apnea during sleep—targets episodes of irregular breathing or apneas, indicating possible upper airway obstruction during sleep. Item 17—daytime fatigue or sleepiness—assesses the long‐term effects of disturbed sleep, such as excessive daytime fatigue, reduced concentration, and impaired daily functioning. Together, these items expand the scope of the instrument to include symptoms highly relevant for patients with tonsil‐related sleep pathology. The revised instrument has a maximum total score of 85. An adjusted score is calculated by dividing the sum by 85 and multiplying with 100, resulting in a score ranging from 0 to 100. The full TOI‐14 and TOI‐17 questionnaires in Swedish are provided in Appendix A in Supporting Information.

In addition to completing the TOI‐14 and the TOI‐17, study participants were asked to fill out the EQ‐5D VAS. The EQ‐5D is a widely used quality of life symptom assessment instrument [11]. The VAS comprises a vertical scale ranging from 0, representing “the worst health you can imagine,” to 100, indicating “the best health you can imagine.” The scale is marked in increments of five, with each level labeled numerically and textually (5, 10, etc.) for clarity. Participants were asked to rate their health, as per today, by both marking directly on the scale and writing the corresponding number. For the TOI‐14 and the supplementary SDB items, missing values were imputed using the group‐specific individual item mean. In the EQ‐5D VAS, only one participant in the control group had missing data. No imputation was performed for missing data in the EQ‐5D VAS.

Postoperative follow‐up questionnaires consisting of TOI‐14, TOI‐17, and EQ‐5D VAS were administered 6 months after surgery to all patients in the case group.

### 2.4 The Translation of TOI‐14 Into Swedish

The original German version of the TOI‐14 [8] was translated into Swedish by two authorized translators and merged into one Swedish version by the authors. This preliminary version was then translated back into German by a new pair of authorized translators. The two translated versions were compared with the original TOI‐14, and a final Swedish version (TOI‐14) was created by the authors. The translation and production of the TOI‐14 followed recommendations for translating and culturally adapting PROMs [12].

### 2.5 Statistical Analyses

Descriptive statistics were used to summarize participant characteristics, including age, sex, surgical indication, baseline EQ‐5D VAS, TOI‐14, and TOI‐17 scores. Means, standard deviations, medians were calculated where appropriate. Statistical analyses were performed in IBM SPSS, except for CFA which was conducted in R (version 4.4.3) using the lavaan package, and EFA which was conducted in R (version 4.4.3) using the psych package.

The psychometric validation strategy was predefined in accordance with international recommendations for PROMs, primarily the COSMIN (COnsensus‐based Standards for the selection of health Measurement INstruments) guidelines—covering reliability, validity, responsiveness, and interpretability [13]. To assess convergent validity, the correlation between TOI‐14/TOI‐17 total scores and self‐rated health measured with the EQ‐5D VAS was calculated using Spearman's rank correlation. Known‐groups validity was assessed by comparing the TOI‐14/TOI‐17 scores of the case group and control group at baseline. The EQ‐5D VAS score was used to compare self‐perceived general health between the case and control group. To assess test–retest reliability, participants in both the case and control groups were asked to repeat the completion of the TOI‐14 and the three additional items included in TOI‐17, 2 weeks following the baseline assessment. The reliability of the TOI‐14 and TOI‐17 in the total study population across 2 weeks was assessed using the intraclass correlation coefficient (ICC), two‐way mixed effect single measures. TOI‐14 and TOI‐17 test–retest reliability was also evaluated separately for the different indications in the case group. An ICC of 0.7 or greater was considered acceptable reproducibility [14]. The responsiveness of the TOI‐14 and TOI‐17 total scores (baseline versus follow‐up after surgery) was analyzed with Wilcoxon signed rank test. The internal consistency of the TOI‐14 and TOI‐17, as well as the original four domains (throat discomfort, general health, resources, and social‐psychological restrictions) and the additional SDB domain was analyzed with Cronbach's *α*. A value > 0.70 was considered acceptable reliability [15]. *p*‐values < 0.05 were considered significant.

To evaluate the underlying structure of the Swedish version of the TOI‐14, a CFA [16] was performed. The model was specified according to the original structure of the instrument, with four latent factors: throat discomfort, general health, resources, and social‐psychological restrictions [8]. An acceptable model includes comparative fit index (CFI) and Tucker–Lewis index (TLI) > 0.90, root mean square error of approximation (RMSEA) and standardized root mean square residual (SRMR) < 0.08 and a nonsignificant chi‐square [17]. As the general health factor consisted of only two items, model identification was ensured by fixing the loading of item 5 to 1 and constraining the variance of the latent factor to 1. Conversely, an EFA [18] was performed for the TOI‐17. A maximum likelihood estimation approach was used, and the model was specified for four latent factors.

### 2.6 Ethics Statement

The study was approved by the Swedish Ethical Review Authority 2022‐09‐15 (Dnr 2022‐03966‐01) and conducted in accordance with the principles of the Helsinki declaration. Written informed consent was obtained from all participants prior to inclusion.

## 3 Results

### 3.1 Demographics

The baseline characteristics including age, sex, indications and type of surgery, TOI‐14 scores, TOI‐17 scores, and EQ‐5D VAS scores at baseline, retest, and at follow‐up are shown in Table 1. There was no difference in sex across the groups, while the mean age was significantly higher in the control group compared to the case group (*p* = 0.014). Despite patients being lost to follow‐up, the proportion of patients in the different surgical indications at 2 weeks and 6 months was largely consistent with baseline.

**TABLE 1:** Baseline characteristics of study population; TOI‐14, TOI‐17, and EQ‐5D‐VAS scores at baseline, retest, and at follow‐up.

	Case group (*n* = 108)	Control group (*n* = 84)
Age (years), mean (SD)	27.1 (7.9)	30.1 (9.1)
Gender (female), *n* (%)	56 (51.9%)	44 (52.4%)
Indication for surgery, *n* (%)		
SDB/tonsil hypertrophy	28 (25.9%)	na
Recurrent tonsillitis	32 (29.6%)	na
Peritonsillitis	22 (20.4%)	na
Chronic tonsillitis	26 (24.1%)	na
Type of tonsil surgery, *n* (%)		
Tonsillectomy	100 (92.6%)	na
Tonsillectomy + adenoidectomy	3 (2.8%)	na
Tonsillotomy	4 (3.7%)	na
Tonsillotomy + adenoidectomy	1 (0.9%)	na
TOI‐14, mean (SD)		
At baseline	39.5 (17.8)	3.9 (4.5)
At retest	34.7 (16.8)^a^	2.9 (4.0)^b^
Six months after surgery	9.9 (13.3)^c^	na
TOI‐17, mean (SD)		
At baseline	50.7 (19.8)	4.9 (5.0)
At retest	36.5 (15.2)^a^	3.1 (3.8)^b^
Six months after surgery	10.9 (12.6)^c^	na
EQ‐5D VAS, mean (SD)		
At baseline	70.5 (19.1)	86.8 (7.7)
At retest	72.0 (19.5)^a^	86.9 (9.2)^b^
Six months after surgery	78.2 (14.9)^c^	na

^a^ *n* = 37.

^b^ *n* = 71.

^c^ *n* = 75.

### 3.2 Missing Data

Some case group subjects were recruited on the day of surgery, preventing these participants from undergoing the retest data collection. The number of missing responses per item was low across all items in the TOI‐14 (*n* = 16, 0.3%), as well as in the SDB items 15–17 (*n* = 14, 1.2%). Cross tabulation of the missing data distribution indicated that data were missing at random, both at the level of individual items and across data collection time points.

### 3.3 Convergent Validity

Both the TOI‐14 and TOI‐17 total scores in the total population (*n* = 192) were significantly correlated with general health (EQ‐5D VAS) at baseline with Spearman rank correlation coefficients of −0.54 (*p* < 0.001) and −0.55 (*p* < 0.001) respectively. In the case group the correlation coefficient was −0,25 (*p* 0.008) and −0.26 (*p* 0.006) for the TOI‐14, and TOI‐17 (*n* = 108).

### 3.4 Known Groups Validity

**FIGURE 2:**
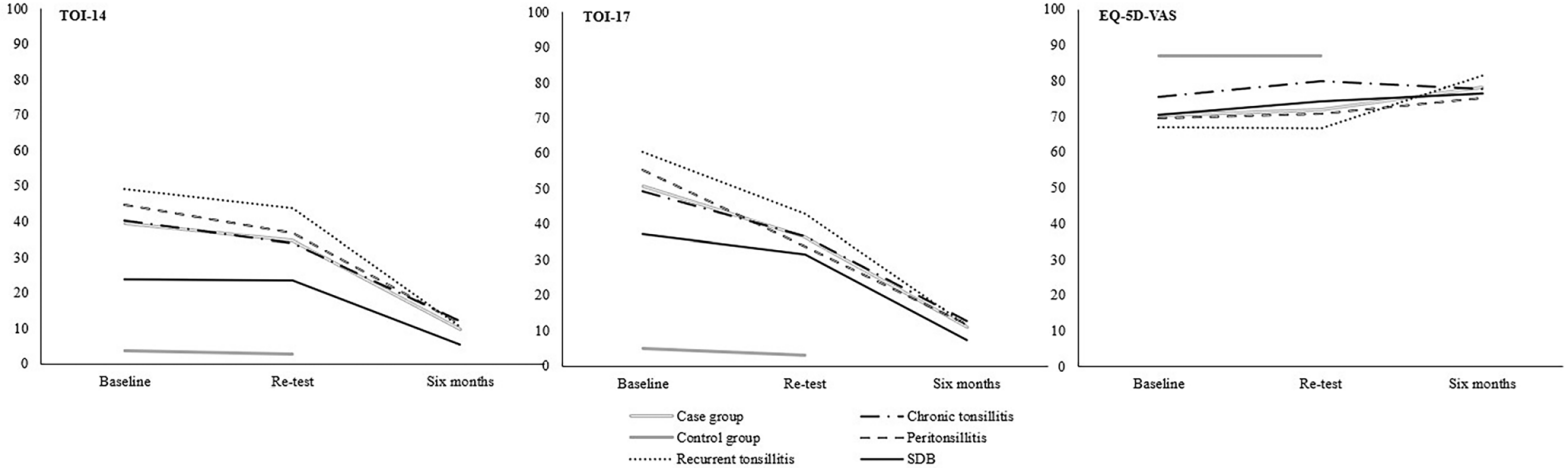
Mean scores for TOI‐14, TOI‐17, and EQ‐5D VAS at baseline, retest, and at follow‐up 6 months after surgery. The number for each group is described in Table 1.

At baseline and retest, subjects in the case group reported significantly higher (*p* < 0.001) total mean scores for both TOI‐14 and TOI‐17, and lower mean EQ‐5D VAS score compared to controls (Table 1 and Figure 2).

### 3.5 Test–Retest Reliability

The test–retest reliability of the TOI‐14 and TOI‐17 scores in the total population (*n* = 192) across 2 weeks was high with ICCs of 0.893 (95% CI 0.845–0.926) and 0.831 (95% CI 0.69–0.90), respectively. In the case group, the TOI‐14 ICCs for the different indications were: SDB 0.81 (95% CI 0.469–0.754), recurrent tonsillitis 0.727 (95% CI 0.291–0.913), chronic tonsillitis 0.867 (0.325–0.98), peritonsillitis—0.070 (−0.704–0.625).

### 3.6 Responsiveness

Patients in the case group showed significant improvement at follow‐up 6 months postoperatively (Table 1), with lower TOI‐14 and TOI‐17 scores and higher EQ‐5D VAS scores compared to baseline (*p* < 0.001), consistent across all surgical indications.

### 3.7 Internal Consistency

Cronbach's *α* for the TOI‐14 and TOI‐17 items in the case group at baseline was 0.88 and 0.816 respectively. All domains of the TOI‐14 were internally consistent in the case group with Cronbach's *α* above 0.7: Throat discomfort (items 1–4) Cronbach's *α* 0.85, general health (items 5–6) Cronbach's *α* 0.82, resources (items 7–10) Cronbach's *α* 0.89, and social‐psychological restriction (items 11–14) Cronbach's *α* 0.90. The additional SDB domain (items 15–17) was also internally consistent in the case group with a Cronbach's *α* 0.85.

### 3.8 Item Scores

**FIGURE 3:**
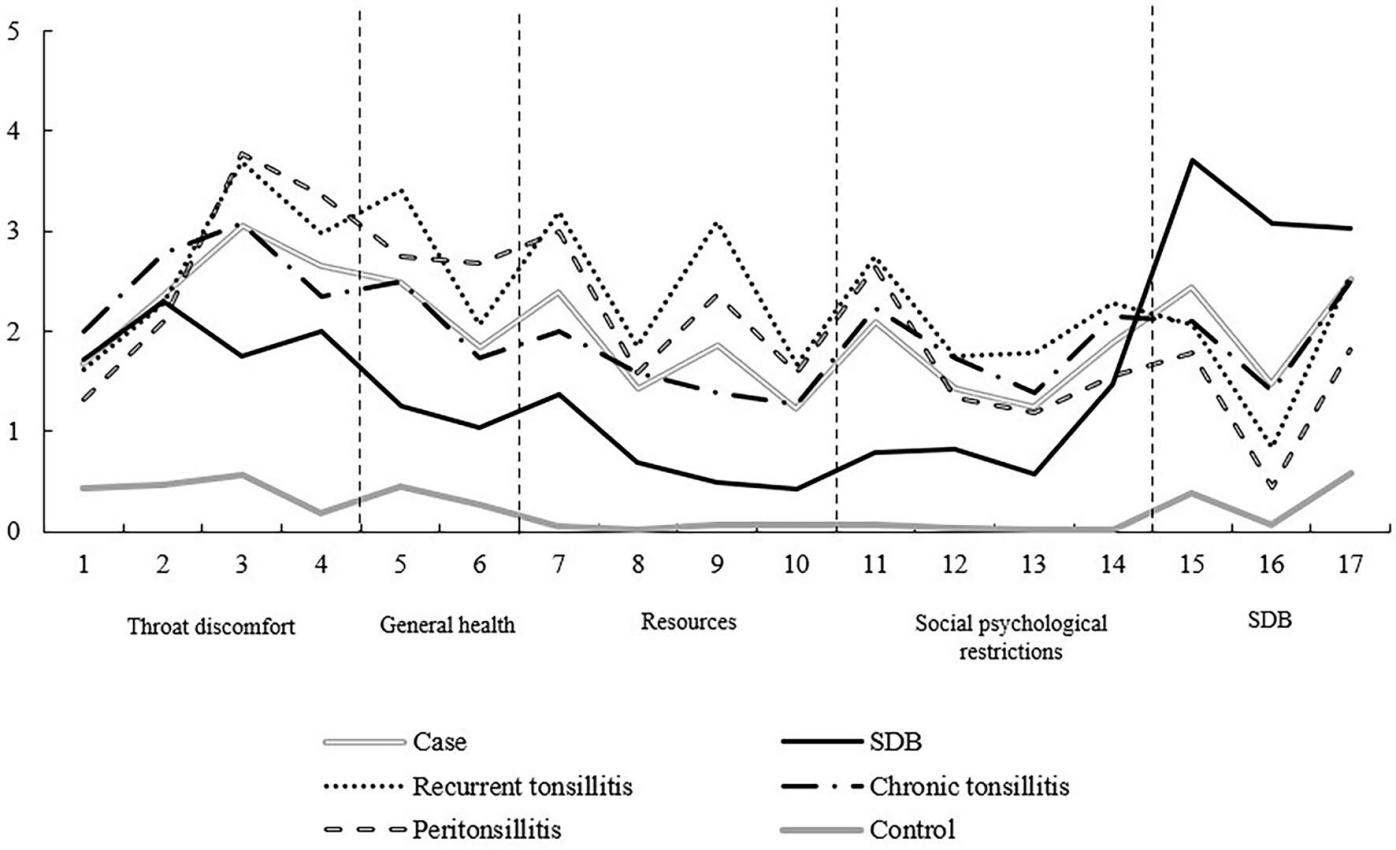
Mean item scores (0–5) of the TOI‐14 and TOI‐17 across all domains, shown for the control group, the case group, and for each surgical indication separately.

The mean TOI‐17 item scores at baseline across surgical indication groups (recurrent tonsillitis, chronic tonsillitis, peritonsillitis, SDB) compared with healthy controls are presented in Figure 3. At baseline, patients with recurrent tonsillitis reported the highest symptom burden across domains (TOI‐17 total mean score = 60), followed by those with peritonsillitis (55) and chronic tonsillitis (49), whereas patients with SDB reported a comparatively lower overall symptom burden (37). In contrast, controls consistently reported near‐minimal symptom levels.

### 3.9 Factor Structure Analysis

**FIGURE 4:**
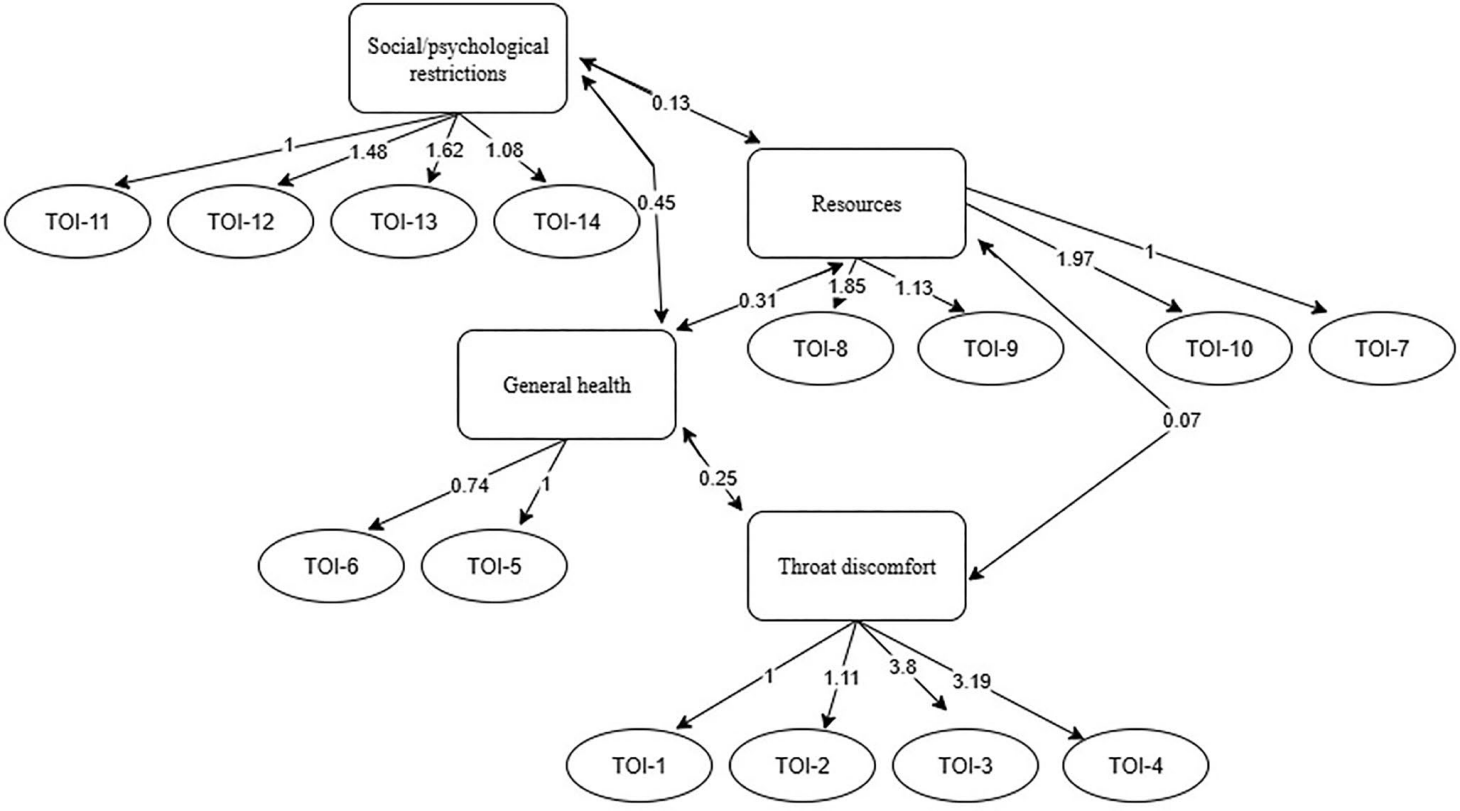
CFA of the Swedish TOI‐14, specifying four latent factors: Throat discomfort (items 1–4), general health (items 5–6), resources (items 7–10), and social‐psychological restrictions (items 11–14). Paths show unstandardized loadings (marker loadings fixed to 1); curved arrows indicate factor correlations; residuals omitted. General‐health variance was fixed to 1 for identification.

The CFA model for TOI‐14 yielded suboptimal fit indices: *χ* ^2^ *p* < 0.05, CFI = 0.828, TLI = 0.783, RMSEA = 0.132, and SRMR = 0.137, indicating poor model fit. As shown in Figure 4, most items loaded as expected on their respective factors. However, the general health factor, comprising only two items, displayed notable cross‐loadings, indicating that these items were not clearly distinct from other domains and may capture overlapping constructs.

**FIGURE 5:**
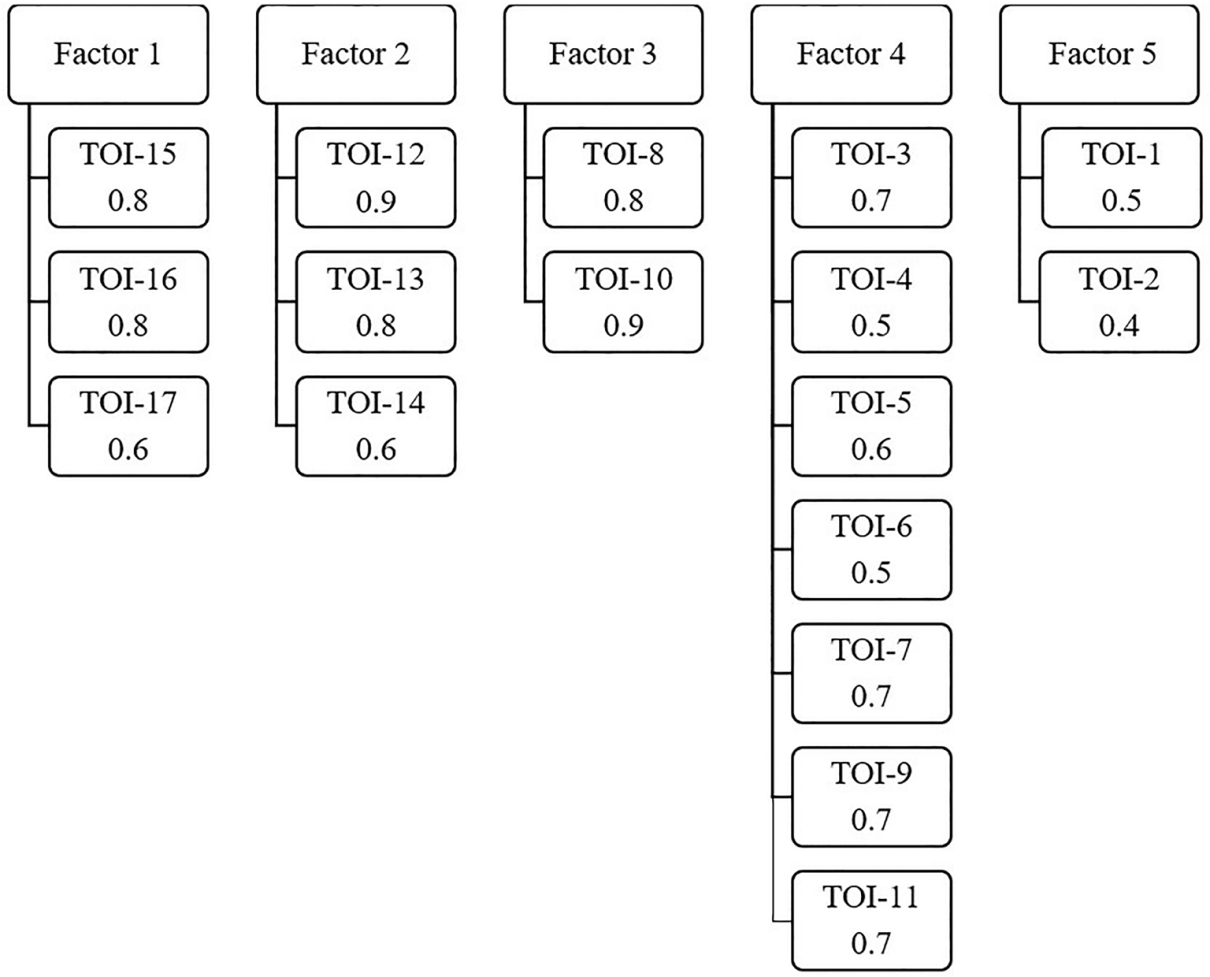
EFA of the Swedish TOI‐17 showing the five‐factor solution with corresponding item loadings. Each column represents a latent factor, and the respective TOI‐17 items with their standardized loadings are displayed beneath.

The EFA scree plot for the TOI‐17 supported a five‐factor solution (Appendix B in Supporting Information). The first factor explained 18% of the variance, followed by 14%, 11%, 10%, and 6% for the subsequent factors, cumulatively accounting for 60% of the total variance. On average, each item loaded onto 1.6 factors. The Chi‐square goodness‐of‐fit test was significant (*p* = 0.017), suggesting a suboptimal fit. However, incremental fit indices were acceptable, with TLI = 0.927 and RMSEA = 0.062, supporting a reasonably good model performance. The factor loading matrix is presented in Figure 5. One factor (Factor 1) emerged comprising the three SDB items (TOI‐17 items 15–17). The factor loadings were 0.8 for item 15, 0.8 for item 16, and 0.6 for item 17.

## 4 Discussion

This study aimed to translate and validate a Swedish version of the TOI‐14 as well as to explore an extended TOI‐17 version including SDB items in a typical Swedish cohort of tonsil surgery patients. The main findings demonstrate that both TOI‐14 and TOI‐17 are reliable, valid, and responsive instruments for assessing patient‐reported outcomes in adults undergoing tonsil surgery. We argue that this holds true even though the CFA of the translated instrument provided a suboptimal fit of the original factor solution. We also found the extended TOI‐17 version to capture sleep‐related symptoms that are not represented in the original instrument, thereby yielding an instrument with broader clinical applicability more suitable to cover all indications in an adult Swedish tonsil surgery population.

### 4.1 Main Findings

Our results are consistent with the original German validation study of the TOI‐14, which reported high internal consistency, test–retest reliability, and construct validity. In line with earlier findings, the Swedish TOI‐14 achieved Cronbach's *α* values well above the accepted threshold of 0.70 across all domains, confirming the robustness of the scale in a new cultural and linguistic setting. Responsiveness of both TOI‐14 and TOI‐17 was strong, with substantial improvements in scores 6 months postoperatively, supporting their sensitivity to clinical change.

The addition of SDB items proved clinically relevant for the intended target group: Swedish adults undergoing tonsil surgery for all indications. By incorporating three targeted SDB items, the extended version demonstrated good reliability and validity, indicating that it can be used to more comprehensively evaluate patients with obstructive symptoms. Clinically, the mean item profiles (Figure 2) highlight the discriminatory capacity of TOI‐14/TOI‐17. Patients with recurrent tonsillitis and peritonsillitis reported the highest levels of throat‐related discomfort across most items. In contrast, patients with SDB demonstrated relatively mild throat symptoms but a distinct peak in items 15–17, reflecting sleep‐related problems. The control group consistently scored near zero across all domains, underscoring the instrument's sensitivity in differentiating symptomatic from asymptomatic individuals. These findings support the construct validity and clinical interpretability of the Swedish TOI‐14 instruments.

We conducted analyses to evaluate the factor structure of the Swedish TOI‐14 and its extended version, TOI‐17. The original development study identified a four‐factor structure representing throat problems, general health, resource use, and social‐psychological restrictions [8]. In contrast, our CFA indicated a suboptimal fit for this model, suggesting limited replicability in the Swedish context. In the original German study, the principal component analysis yielded a first eigenvalue of 5.9, explaining 42.2% of the variance, with three additional components (eigenvalues 1–2) contributing another 29.9%, for a cumulative explained variance of 72.2% [8]. A later normative validation in a German population (*n* = 1000) identified a three‐component structure—physiological, psychological, and socioeconomic—explaining 73% of the variance, with all factor loadings above 0.5 [9]. In our Swedish validation, the CFA of the TOI‐14 showed moderate but insufficient fit indices. Most items loaded as expected, but the general health domain, consisting of only two items, exhibited notable cross‐loadings, implying overlap with other domains and a less distinct construct. This pattern aligns more closely with the three‐component solution reported in the normative German population study than with the original four‐factor model, indicating that the latter may be less stable across populations. Such divergence may reflect linguistic nuances, cultural differences in symptom perception, or sample size limitations. EFA of the TOI‐17 supported a five‐factor solution explaining 60% of the total variance. A distinct fifth factor emerged from the three added SDB items (loadings 0.6–0.8), indicating strong internal consistency and conceptual coherence. Although patients with tonsil‐related SDB may have concomitant inflammatory tonsil symptoms, these conditions are often partially independent. This may explain why patients with SDB reported comparatively low TOI‐14 scores but distinct elevations in the added SDB items, supporting the conceptual separation observed in the factor analysis. Overall, the Swedish validation partly diverged from the original German models, mainly due to the weaker differentiation of the general health domain, while the new SDB items formed a robust and clinically meaningful additional dimension, consistent with the increasing recognition of sleep‐related morbidity in tonsil surgery patients.

### 4.2 Strengths and Limitations

Strengths of this study include its prospective, multicenter design involving both university and regional hospitals. The included patients display demographic characteristics that are closely comparable to those reported in previous large national cohorts [2, 7]. In addition, the use of both disease‐specific and generic quality of life instruments (EQ‐5D VAS) provides a solid foundation for assessing convergent validity. The discriminative ability of TOI‐14 and TOI‐17 across diagnostic subgroups, and sensitivity to postoperative improvement, shows their potential for use in future clinical decision‐making and in long‐term monitoring of outcomes.

Several limitations should be acknowledged. Although we initially aimed for a larger cohort, recruitment was challenging as it relied on voluntary physician engagement during routine clinical practice. Inclusion was discontinued once recruitment declined, which may have reduced statistical power. Attrition at retest and follow‐up reduced sample sizes for certain analyses, which may have affected the precision of reliability and responsiveness estimates.

The lack of stringent, standardized surgical definitions for peritonsillitis and SDB represents an additional limitation. Data from the SQTS (2023–2025) indicate that approximately 30% of tonsil surgeries for peritonsillitis were performed *à chaud*, that is, during an acute infection. However, the SQTS does not capture information on the number of prior peritonsillitis episodes. In Swedish clinical practice, supported by local data reported by Agerhäll et al., interval tonsillectomy is commonly offered to adult patients after a second episode of peritonsillitis, particularly when at least one additional indication is partially present [19].

For SDB, national guidelines recommend objective diagnostic confirmation with at least overnight cardiorespiratory polygraphy [20]. Such data were not available in the present study, and adherence to this recommendation among Swedish ENT surgeons before tonsillectomy—when clinical presentation and history are considered convincing—cannot be assessed. Although the inclusion of SDB‐related items added clinical value, these items—especially those addressing snoring and nocturnal breathing disturbances—may primarily rely on observations by partners or family members rather than the patient alone. Consequently, responses may be influenced by social context or living arrangements, which may change independently of surgical intervention. This limitation is inherent to many sleep‐related PROMs and should be considered when interpreting TOI‐17 scores, particularly when analyzed in isolation.

Subgroup analyses were limited by small sample sizes. For instance, the relatively small number of both peritonsillitis and SDB patients limits firm conclusions regarding validity in these subgroups. The peritonsillitis subgroup included 22 patients at baseline, of whom only 15 provided follow‐up data. In particular, the peritonsillitis subgroup also showed poor test–retest reliability, which we interpret to be a consequence of clinical practice. In Sweden, the common approach is to manage peritonsillitis with antibiotics and surgical drainage under local anesthesia, while tonsillectomy is generally considered only after two episodes and usually performed once the acute infection has resolved. As a result, a recent episode may therefore disproportionately influence TOI‐14 responses at baseline and retest, which challenges the relevance of a six‐month retrospective questionnaire in this subgroup, resulting in considerable intragroup variability and consequently a low ICC.

Finally, we were unable to fully assess overall construct validity, as no other disease‐specific PREM/PROM instrument currently exists for this patient group in Swedish. Cronbach's α values exceeding 0.9, while demonstrating strong internal consistency, may also indicate redundancy among items.

## 5 Conclusion

The Swedish TOI‐14 and TOI‐17 are valid, reliable, and responsive instruments for assessing disease‐specific quality of life in adults with tonsil disease. The extended version TOI‐17 adds important coverage of sleep‐related symptoms, increasing clinical relevance. Incorporating these PREMs/PROMs into clinical practice and the SQTS can enhance patient‐centered care and inform evidence‐based surgical decision‐making.

## Funding

The authors have nothing to report.

## Conflicts of Interest

The authors declare no conflicts of interest.

## Supporting information


**Appendix A.** The Swedish version of the TOI‐14. The TOI‐17 additionally includes the three supplementary SDB items (highlighted in gray).
**Appendix B**. Scree plot and parallel analysis supporting a five‐factor solution for the TOI‐17.

## Data Availability

The data that support the findings of this study are available on request from the corresponding author. The data are not publicly available due to privacy or ethical restrictions.
