# Supplementary material for: Validation and Clinical Adaptation of a Swedish Version of the Tonsil Outcome Inventory‐14 Questionnaire
Source: Laryngoscope Investig Otolaryngol. 2026 Feb 18;11(1):e70359. doi: 10.1002/lio2.70359 (PMC12916258; doi:10.1002/lio2.70359)
Supplement: Supplementary file 1 — Appendix A. The Swedish version of the TOI‐14. The TOI‐17 additionally includes the three supplementary SDB items (highlighted in gray). Appendix B. Scree plot and parallel analysis supporting a five‐factor solution for the TOI‐17. [file LIO2-11-e70359-s001.docx]

**Supplementary material**

*Appendix A*

The Swedish version of the TOI-14. The TOI-17 additionally includes the three supplementary SDB items (highlighted in grey).

| För att kunna bedöma hur svåra de enskilda problemen är markera lämplig siffra vid varje enskild fråga med ett kryss. | **Inga**  **problem** | **Obetydliga**  **problem** | **Lindriga problem** | **Måttliga**  **problem** | **Svåra problem** | **Problemen är så illa de kan bli** |
| --- | --- | --- | --- | --- | --- | --- |
| 1. Torr i halsen | 0 | 1 | 2 | 3 | 4 | 5 |
| 2. Segt slem i svalget | 0 | 1 | 2 | 3 | 4 | 5 |
| 3. Ont i halsen | 0 | 1 | 2 | 3 | 4 | 5 |
| 4. Svårt att svälja | 0 | 1 | 2 | 3 | 4 | 5 |
| 5. Sjukdomskänsla | 0 | 1 | 2 | 3 | 4 | 5 |
| 6. Nedsatt fysisk prestationsförmåga | 0 | 1 | 2 | 3 | 4 | 5 |
| 7. Upprepade läkarbesök | 0 | 1 | 2 | 3 | 4 | 5 |
| 8. Kostnader för läkarbesök | 0 | 1 | 2 | 3 | 4 | 5 |
| 9. Upprepade antibiotikabehandlingar | 0 | 1 | 2 | 3 | 4 | 5 |
| 10. Kostnader för läkemedel | 0 | 1 | 2 | 3 | 4 | 5 |
| 11. Mina halsbesvär orsakar så mycket frånvaro från arbete eller skola att det medför problem | 0 | 1 | 2 | 3 | 4 | 5 |
| 12. På grund av halsbesvären går jag mindre ofta på offentliga evenemang och tillställningar | 0 | 1 | 2 | 3 | 4 | 5 |
| 13. På grund av halsbesvären träffar jag vänner/anhöriga mindre ofta | 0 | 1 | 2 | 3 | 4 | 5 |
| 14. På grund av halsbesvären känner jag mig nedstämd | 0 | 1 | 2 | 3 | 4 | 5 |
| 15. Snarkar högljutt under sömn | 0 | 1 | 2 | 3 | 4 | 5 |
| 16. Oregelbunden andning eller andningsuppehåll (apné) under sömn | 0 | 1 | 2 | 3 | 4 | 5 |
| 17. Dagtrötthet eller dagsömnighet | 0 | 1 | 2 | 3 | 4 | 5 |

*Appendix B*

Scree plot and parallel analysis supporting a five-factor solution for the TOI-17.

**
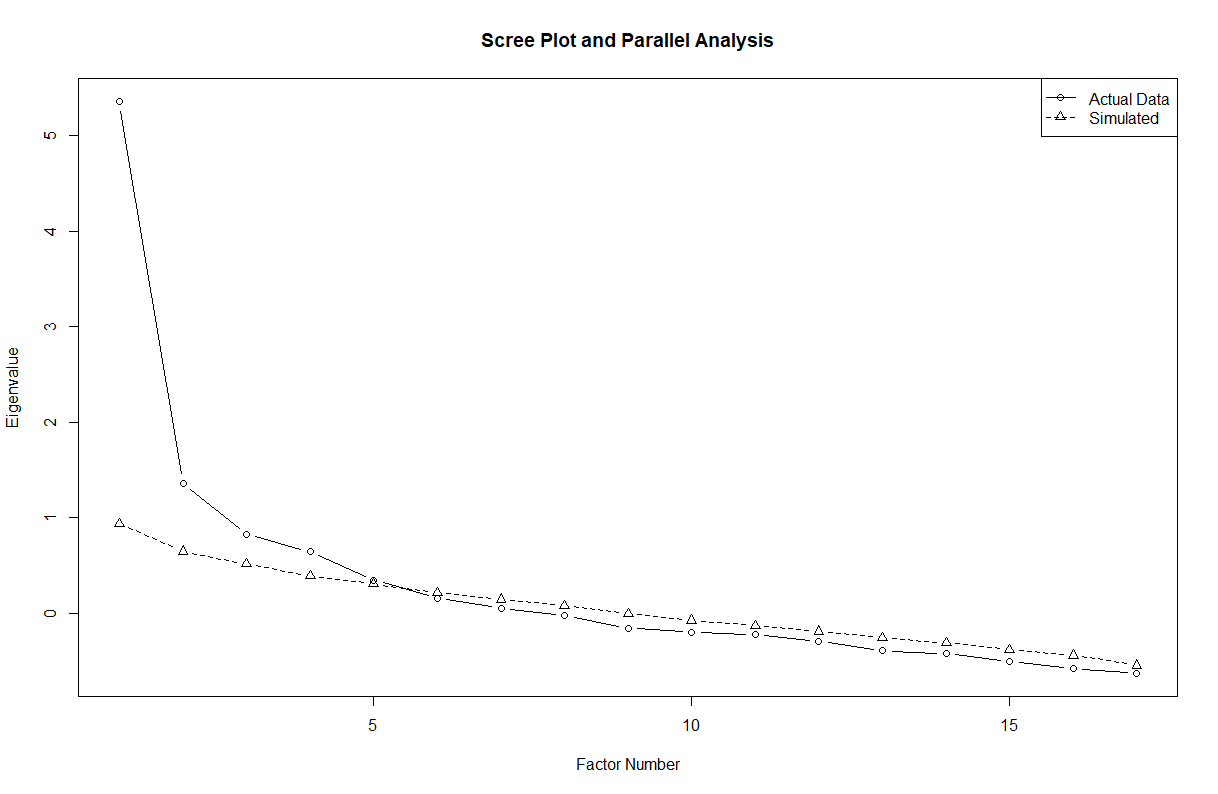
**
